# The Influence of Parental Dietary Behaviors and Practices on Children’s Eating Habits

**DOI:** 10.3390/nu13041138

**Published:** 2021-03-30

**Authors:** Lubna Mahmood, Paloma Flores-Barrantes, Luis A. Moreno, Yannis Manios, Esther M. Gonzalez-Gil

**Affiliations:** 1 Growth, Exercise, Nutrition and Development (GENUD) Research Group, Instituto Agroalimentario de Aragón (IA2), University of Zaragoza, 50009 Zaragoza, Spain; lmahmood400@gmail.com (L.M.); pfloba@unizar.es (P.F.-B.); esthergg@ugr.es (E.M.G.-G.); 2 Instituto Agroalimentario de Aragón (IA2), 50009 Zaragoza, Spain; 3 Instituto de Investigación Sanitaria de Aragón (IIS Aragón), 50009 Zaragoza, Spain; 4 Centro de Investigación Biomédica en Red de Fisiopatología de la Obesidad y Nutrición (CIBERObn), Instituto de Salud Carlos III, 28040 Madrid, Spain; 5 Department of Nutrition and Dietetics, School of Health Science & Education, Harokopio University, 17671 Athens, Greece; manios@hua.gr; 6 Institute of Agri-food and Life Sciences, Hellenic Mediterranean University Research Centre, 71410 Heraklion, Greece; 7 Department of Biochemistry and Molecular Biology II, Instituto de Nutrición y Tecnología de los Alimentos, Center of Biomedical Research (CIBM), Universidad de Granada, 18071 Granada, Spain

**Keywords:** parents, dietary intake, feeding practices, children, family meals, breakfast, snacking habits

## Abstract

Poor dietary habits established during childhood might persist into adulthood, increasing the risk of developing obesity and obesity-related complications such as Type 2 Diabetes Mellitus. It has been found that early modifications in eating habits, especially during childhood, might promote health and decrease the risk of developing diseases during later life. Various studies found a great influence of parental dietary habits on dietary behaviors of their children regardless of demographic characteristics such as gender, age, socioeconomic status and country; however, the exact mechanism is still not clear. Therefore, in this review, we aimed to investigate both parents’ and children’s dietary behaviors, and to provide evidence for the potential influence of parents’ dietary behaviors and practices on certain children’s eating habits. Family meals were found to contribute the most in modeling children’s dietary habits as they represent an important moment of control and interaction between parents and their children. The parental practices that influenced their children most were role modeling and moderate restriction, suggesting that the increase of parental encouragement and decrease of excessive pressure could have a positive impact in their children’s dietary behaviors. This narrative review highlights that parental child-feeding behaviors should receive more attention in research studies as modifiable risk factors, which could help to design future dietary interventions and policies to prevent dietary-related diseases.

## 1. Introduction

Obesity is a complex condition influenced by both genetic and environmental factors [1]. Dietary intake has been linked with obesity in terms of volume, composition, meals’ frequency, snacking habits and diet quality [2]. Additionally, there is indication that children are likely to maintain their dietary habits into adulthood [2]. Thus, understanding children’s eating habits is very important in terms of children’s health [3]. There are some factors that could influence children’s eating habits such as the home food environment, as well as the social environment, contexts where perceptions, knowledge and eating habits are established [4]. However, parental dietary patterns seem to affect children most, as parents are the ones who shape the home food environment, influence how a child thinks about food, and, accordingly, start forming their own food preferences and eating behavior [4].

Out of the dietary habits, family mealtime becomes the main social context in which children can eat with their parents, who are considered as their main role-models [5]. Sharing meals with children, having breakfast together regularly and encouraging children to have healthy snacks with moderate restrictions have shown positive impacts on children’s dietary behaviors [6]. Furthermore, one review study evaluated these practices and found that they were associated with higher consumption of dairy products, fruits and vegetables (FV), along with healthier breakfast patterns among children [7]. Also, the same review stated that encouragement practice gives children a chance of making decisions, whereas the moderate restriction practice help parents to imply clearer instructions to their children. Therefore, it was recommended to use a combination of the two practices, so that both parents and children would have the ability to contribute to determining food choices [8]. In this narrative review, we focus on the effect of parental dietary habits on children’s eating behaviors, including family meals, breakfast routine and snacking habits.

## 2. Method for Literature Search

Serial literature searches for articles of interest were performed between August and December 2020. PubMed, Scopus, Education Resources Information Center (ERIC), Science Direct and Google scholar databases were searched using the following keywords: “parents”, “dietary intake”, “feeding practices”, “children”, “family meals”, “breakfast”, “snacking habits”, “food choices”, “food consumption”, “role model”, “diabetes”, “parenting style”, “behavior”. We included both original researches and review articles published between 2000 and 2020. Studies were eligible if they were published in English and included preschoolers (ages 2–5 years), or school-age children (ages 6–13 years). A total of 2590 studies were identified, 508 duplicates were removed, 455 related titles were chosen, 92 articles met the inclusion criteria and 83 studies were included in this review.

## 3. Definitions

“Eating habits” can be defined as the conscious and repetitive way a person eats, and this includes what types of food are eaten, their quantities and timing of consumption, in response to cultural and social influences [9]. On the other hand, “eating behaviors” have been considered as a group of actions starting from a simple food chewing to food shopping, food preparation and food policy decision-making [10]. Food patterns or dietary patterns refer to the quantity, quality and variety of foods and beverages consumed as well as the frequency with which they are habitually consumed, and it refers to the diet as a whole [10]. A balanced diet is characterized by high intake of fresh FV, whole grains, legumes, nuts, fiber, polyunsaturated fatty acids and low in both refined grains as well as saturated fatty acids [11]. However, guidelines may differ in their recommendations regarding the consumption of processed meat and dairy products, probably relating to the national food culture, sustainable food choices and food safety [11].

## 4. Children’s Eating Habits

Dietary habits from childhood track into adulthood, so understanding children’s eating habits is very important in terms of children’s health [12]. Nutrition is the main factor of interaction between parents and children, especially during the first year of life, starting by breastfeeding [12]. By the end of the first year of life, children start learning to feed themselves and make the transition to the family diet and meal patterns [12]. A review study that assessed both national and international research articles on child nutrition and eating behaviors concluded that as children switch to the family diet, recommendations from parents address not only food, but also the eating context, which refers to the immediate environment of each eating occasion [12]. Moreover, the same study suggested that a variety of healthy food items provided to children can promote their diet quality and food acceptance [12].

A study across 11 countries suggested that the nutritional status of children from birth to the age of 2 years was positively associated with dietary variety [13]. Furthermore, exposures to FV in early childhood have been associated with higher acceptance of these foods at later ages [13]. A longitudinal study of 120 2-year-old children and their parents followed for 9 years found that around 25% of children experienced some eating problems such as being hesitant to try new foods or insist on a limited number of food items (no variety), concluding that those problems may lead them to become picky eaters [14].

However, children with eating problems (i.e., picky eaters, meal skippers) may be at risk for behavioral problems, as well as impaired growth and development [14], whereas repeated exposure was found to be the main way for children to recognize the food. Thus, parents are advised to keep introducing food items more than once, and to avoid getting discouraged or giving up [12].

## 5. Home Food Environment

The home food environment includes the availability and accessibility of food, as well as other factors such as frequency of eating out, and parents’ perception of food costs [15]. In addition, the home food environment was found to have remarkable effects on eating behaviors of parents and their children as most of the food consumed is stored and prepared at home [15]. Although children are likely to be influenced by their home food environment and the community, they may have limited control over it [16]. Results from the Quebec Longitudinal Study of Child Development, which included 1492 children, found that children who had a better family environment, i.e., less family pressure to eat, had low levels of soft-drinks consumption (unstandardized β = −0.43, *p* < 0.001, 95% confidence interval (CI), –0.62 to −0.23), and high level of fitness (unstandardized β = 0.24, *p* < 0.001, 95% CI, 0.12–0.36) [16]. In the same vein, the baseline survey of the Identification and prevention of Dietary- and lifestyle-induced health Effects in Children and infants (IDEFICS) study, which included 1435 families from eight European countries, found that home food environment plays a stronger role in shaping children’s intake of healthy foods than unhealthy foods, especially for younger children [17].

As previously mentioned, the home food environment determines what kind of foods are available and accessible to children [15]. While availability and accessibility are often merged into a single construct, the content map presented in Vaughn’s study [18] considered them separately because they may have differential effects on children’s diet and eating behaviors. Accordingly, these diverse definitions could explain the differences found in the results of studies. Availability is related to the physical presence of food [18], whereas accessibility refers to parental actions to control how easy or difficult it is for children to access food by themselves or with limited assistance [18]. A review about the availability and accessibility of FV at home found that both availability and accessibility were associated with FV consumption among children and adolescents and inversely associated with children’s total energy and fat intake [19].

Besides, low consumption of nutrient-poor, energy-dense food items, like sugar-sweetened beverages, cookies, packed snacks, food high in saturated/transfat, simple sugars and sodium, were noticed when these items were and were not available at home [19]. However, low-income families seem to have low access to healthy foods and possibly greater access to fast food due to dietary costs, which could explain some of the relationships between Socioeconomic Status (SES) and nutrient density of consumed foods [19].

Frequency of eating out is one of the dietary habits that are most influenced by the household environment [20]. Ready-to-eat and out-of-home (OH) foods include vending machines, take-away, cafes, restaurants, supermarkets and convenience stores [20]. Nowadays, families seem to prepare less food at home and spend more money on foods prepared away from home [20], and food prepared OH tends to be more energy-dense than food prepared at home, particularly in terms of fat and sugar content [20]. In addition, focus groups among the urban community in the US found that parents desire easy, convenient and tasteful meals that are culturally appropriate and low-cost, while some families may believe that food eaten out is lower in cost and tastier [21]. These beliefs would encourage parents to eat out and thus perpetuate the cycle of decreased home-prepared meals. Consequently, children may have less opportunities to learn culinary skills, have access to healthy diet, or reinforce healthy eating habits [21]. Cross-sectional data from the UK National Diet and Nutrition Survey Rolling Program of 4636 children and adolescents aged 1.5–18 years showed that consuming food prepared outside the home was associated with a greater intake of foods with high levels of fat and sugar in children [20]. Also, a systematic review documented the nutritional characteristics of eating away from home and its relations with the diet quality and energy intake. The results of this review concluded that eating outside the home is associated with lower diet quality and micronutrients intake, like vitamin C, Fe and Ca. However, the conclusion needed further confirmation as the review was based on studies from national surveys from Belgium and the United States only [22]. Similar results were obtained in a cross-sectional study conducted in Japan among 4258 caregivers, where children with obesity had a lower frequency of shared home-made meals, after adjusting for confounding factors.

However, validity and reliability of the questionnaire used to assess the frequency of cooking were not examined [23]. Unfortunately, these studies have only considered the effect of eating out without concerning the effect of ready-to-eat (unhealthy) meals prepared at home.

## 6. Parenting Styles and Feeding Practices

In the literature, parenting styles have been defined as psychological constructs representing the more general interactions between parents and children, whereas parental feeding practices includes specific rules or behaviors used by parents to control when, what and how much their children eat [24,25].

It has been previously stated by Horst and Sleddens [26] that according to Baumrind’s taxonomy, parenting styles have been divided into three categories: authoritarian, permissive and authoritative. Whereas authoritarian styles are highly demanding but less responsive, permissive styles include less demanding but high responsiveness, and authoritative styles present both demanding and responsive [26].

Studies examining the direct role of parenting styles on children’s eating behaviors are limited. However, a recent review of the evidence found that less parental monitoring was presented in the permissive style, whereas more restrictive food and high pressure on children to eat were linked to authoritarian parenting style. On the other hand, preferable parental monitoring of the child’s food intake was associated with the authoritative parenting style [27]. Another two systematic reviews concluded that children tend to eat more healthily with a healthy body mass index (BMI) if they raised in authoritative households. However, the effects of these generic parenting styles were generally indirect and weak [28,29].

One review critically summarized previous research on parental feeding practices and found that role models can play a really important part in shaping children’s eating habits. Therefore, role modeling behaviors were recommended for parents such as: providing healthy foods, modeling healthy eating and increasing encouragement to eat healthy foods [30]. Results from a study that used the Parental Feeding Style Questionnaire (PFSQ), which included 100 children (aged 2–5) in Hong Kong, showed that encouraging children to consume a variety of foods was associated with healthier eating behaviors, like meal frequency, better food choices and higher intake of fruits (Odd Ratio (OR) = 1.357; 95% confidence interval (CI) = 1.188 to 1.551) and vegetables (OR = 1.335; 95% CI = 1.128 to 1.579) [31]. Whereas, using foods as rewards could increase the child’s preferences for these food items. Thus, using unhealthy foods as rewards may promote children’s consumption of unhealthy energy-dense palatable foods [31]. Likewise, a cross-sectional study conducted in 17 primary schools in Dunedin city in New Zealand found that through a good parental role modeling, higher parental diet quality was associated with lower consumption of cakes, chocolate, biscuits and savory dishes in children [32]. A cross-sectional study included 13,305 children in nine European countries and found associations (OR between 1.40 and 2.42, *p* < 0.02) between parental role modeling of healthful foods with children’s dietary habits, food intake and preferences for fruits and vegetables [33].

The results of these studies highlighted the importance of parental modeling in terms of their dietary behaviors and food choices on the diet of their children. However, parental role modeling studies have employed different methods, with varying validity, to measure children’s dietary intake, such as 24 h dietary recalls, food frequency questionnaires, parent report of child dietary intake and child report of parental role modeling. This could explain why correlations between parent and child reports for these studies have also been mixed, whereas studies that have utilized both parent and child report are very limited.

A review study summarizing previous results on parental strategies and practices concluded that a “moderate restriction” could be beneficial as children of moderately restrictive parents were found to consume fewer calories, eat more fruits, and eat less fatty snacks and sweets [34]. Besides, the “prompting and encouragement” feeding practice made by parents could help their children to have healthier dietary habits [34]. The term “moderate restrictions” indicates a careful use of restrictions by parents in which unhealthy food items were gradually decreased and limited rather than being strictly forbidden, whereas the word ‘encouragement’ refers to the situation when parents offer more types of food with positive messages, but at the same time, children can still make decisions in combination with their parents [31].

On the other hand, restricted parental feeding practice seemed to be related to overeating, especially among preschool-age children [35]. One longitudinal study assessed the maternal influences on picky eating behaviors and diet of 173 9-year-old non-Hispanic white girls [36]. The results of this study suggested that with mothers who were less likely to pressure their children to eat, their children were less likely to be picky eaters or overweight [36]. While, when parents highly restrict energy-dense foods from their children’s diet hoping children choose healthful alternatives, children usually increase their desire for it and start to eat when they are not hungry [34]. Therefore, various research studies discourage pressuring practice as it can create a negative family eating environment and make children pickier eaters [37,38].

Evidence suggests that high involvement and role-modeling practices are more favorable for supporting positive food-related behaviors, especially among young children. But unfortunately, these studies cannot be taken as proof of causality. Thus, long-term studies are needed to determine the causal link between parental feeding practices and children’s eating habits.

Household food rules is another factor which is usually established by parents to guide youth behaviors and achieve goals for their growth [39]. To explain further, for example, both “limited fast food” and “limited portion sizes at meals” were significantly linked with improved food consumption and weight status [39]. Whereas a rule of “no fried snacks” was positively associated with percent body fat (PBF), however, the link between fat intake, snacking and excess weight was unclear as snack foods are often grouped as one item (e.g., chips, candies, ice cream and cookies) [39]. Besides, the “no snacking while watching television” rule was found to be an effective one as children tend to eat more when they are distracted and eating while watching TV also prolongs the eating period [39]. In a School of Public Health Project, Eating and Activity over Time (EAT) researchers found that children in families who watch TV while eating meals had a lower-quality diet than the children of families who turned the TV off during meals [40]. In the same study, children who watched TV while eating family meals seemed to consume fewer grains and vegetables, and more soft drinks, than those who did not watch TV. Similar results were also found among Australian children in which watching TV was associated with the consumption of energy-dense foods and drinks [41]. However, these studies do not definitively prove direct causal effects of household food rules on unhealthy food preferences and overall unhealthy diet.

## 7. Parental Dietary Behaviors Influence on Children’s Eating Habits

**Figure 1:**
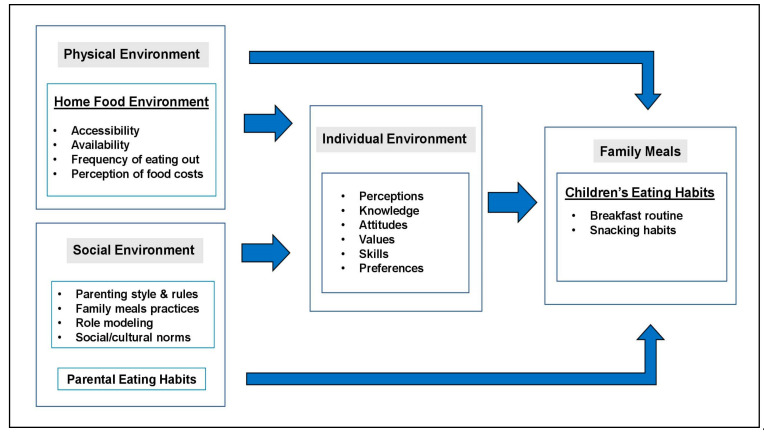
Summary of home/family-related determinants of children’s eating habits.

Dietary preferences are formed by a combination of a complex interplay of genetic, familiar and environmental factors. However, parents seemed to have a high degree of control in modeling their children’s eating behaviors [42]. During the first year of life, children’s dietary patterns undergo a rapid evolution since parents are the ones who select the foods of the family and serve as models of eating. Thus, children tend to imitate their parents’ behaviors as well as eating habits [42]. As illustrated in Figure 1, children’s eating behaviors are affected by social, physical and intra-individual factors. In the family environment, parents establish more than 70% of their children’s dietary behaviors by their own intake and the methods followed to socialize their children [42]. To systematically assess the effect of parental dietary patterns on children, several studies have been revised, which summarize how parental eating habits and feeding styles have been significantly associated with children’s eating behaviors, food preferences, intake and consumption (Supplementary Table S1).

Parental dietary behaviors refer to the passive processes that influence their children’s dietary behaviors and food environment [43]. Various cross-sectional studies have indicated the close similarity between parents and children in the intakes of some healthy and unhealthy foods and beverages, as well as dietary composition, especially when more meals are eaten together [44,45,46,47,48]. Although this association has demonstrated that parents’ dietary behavior might influence children’s intake, these studies cannot be used to conclude causality. Therefore, the process by which parents affect their children’s food intake remains largely unclear.

Four focus groups were conducted in Belgium among parents and caregivers showing that the influence of parental practices differs by age. The younger the child, especially at preschool age and first years of primary education, the stronger the role of parental practices [43]. The same study found that children may consider parents’ norms and perceptions as a reference for what is appropriate to consume [43].

Various cross-sectional studies found showed a significant positive association and substantial correlation between children’s and parent’s intake of various foods [49,50,51]. Thus, parents’ eating behaviors have proven to be a part of the whole process of establishing and promoting healthy or unhealthy dietary patterns among children and adolescents [42]. A Parent Mealtime Action Scale (PMAS) was developed among 439 fathers and 541 mothers in the USA to examine the dimensions of mealtime behaviors used by parents on children’s diet and weight status. The results showed that parents could be influenced by their environment and culture, which may also affect their food choices, suggesting that their children’s dietary patterns and nutritional status may also be altered accordingly [52]. Whereas the same study found that obliging a child to accept healthy food through giving advice only, without eating it themselves, is a dead end in nutrition education [52].

Previous studies concluded that parents’ influence is thought to be strongest during childhood, especially in early ages, when parents act as role models, enforcers and providers. Therefore, intervention programs should consider what parents consume as well as the parental influence in terms of what parents feed their children and how they feed them.

### 7.1. Family Meals

Family meal has been defined as a meal being shared with family members or when one, or both, of the parents are present [53]. There are differences when analyzing the frequency of family meals: some considered it as having ≥3 and others ≥5 family meals taken weekly [53]. Thus, the lack of specificity and consistency in measuring, analyzing and defining family meals makes it difficult to come up with definite results and to compare results [53].

A systematic review [54] that focused on the effects of family meal frequency and psychosocial consequences in youth concluded that more frequent family meals were inversely associated with disordered eating, violent behaviors and depression in children. Additionally, in the same review, it was found that more frequent family meals were positively associated with an increased self-esteem among children [54]. It is agreed that family meals represent an important moment of both control and interaction in the family [55]. A study of family mealtime characteristics of Australian families with children aged 6 months to 6 years old showed that parents place high value on mealtime when they share meals with their children, which helps children to promote healthy eating behaviors. An important strength of this study was the reliable survey measures, but the used online, self-report surveys can be affected by respondent interpretation bias [55]. The presence of parents during mealtime has been linked to decreased meal skipping and increased consumption of dairy products and FV [43]. Correspondingly, results of the Next Generation Health Study in the US showed a higher FV consumption among children whose parents were eating the same food items and sharing their meals with them. This study included a large, nationally representative and generalizable sample; however, the self-estimation and self-report assessment were susceptible to recall bias [56].

In Scotland, a cohort of young children followed-up for 10 years suggested that determining the characteristics of family mealtime practices is needed to increase diet quality and improve children’ eating behaviors, such as reduced access to TV viewing during meals, portion sizes, sitting at a table, besides social engagement between parents and children [57]. Similarly, the Quebec Longitudinal Study of Child Development investigated the effect of frequent family meals on children, and results showed that children who had a better family meal environment at the age of 6 years had lower levels of soft-drinks consumption and higher levels of fitness when they reached 10 years [16]. In the same vein, a Harvard cohort study found that children who eat together with their parents are twice as likely to eat their five servings of FV compared to families who do not share their meals. Moreover, in the same study, family meals seemed to help parents to perform as role models and be considered as an example of polite table manners and healthy eating habits [58]. In addition, results from the same study also showed that shared meals seem to help in childhood obesity prevention as children tend to eat less when they eat in the presence of their parents [58]. Participants in this study were children of nurses, hence, they all came from highly educated families compared to the general population [58].

One meta-analysis concluded that higher frequency of shared family meals in children and adolescents was significantly associated with a normal body weight and healthier dietary habits when they shared family meals 3 or more times per week [59]. Additionally, home cooking and shared family meals have been considered as a key strategy to promote healthy dietary habits and prevent obesity among children [60,61]. A family meals-focused randomized controlled trial in 160 families of 12-year-old children followed-up for about 5 years. Data were collected at baseline, post-intervention and follow-up, and results indicated that promoting healthy shared family meals could lead to a moderate reduction in excess body weight, especially among young children.

Despite the rigorous design, quality measurement and strong theoretical framework used in this study, the generalizability of study findings is limited [61], while engagement in family meals has been considered as the simplest and easiest independent intervention that could be applied to establish a healthy family environment [61]. Therefore, eating environment should be taken into account as it usually affects family communication, parents and children interactions, what kind of food is served, how much is eaten at meals and frequency and lengths of meals. However, it seems that the specific mechanisms of how family mealtimes influence children’s nutritional outcomes are yet unclear and should be investigated.

### 7.2. Breakfast Routine

“Breakfast” refers to the first meal of the day, or a meal often eaten in the early morning [62]. The findings from the “Anthropometry, Intake and Energy Balance” (ANIBES) Study [62] reported that around 85% of the Spanish population (9–75 years) were regular breakfast consumers, although one in five adolescents were breakfast skippers. It has also been found in the same study that breakfast provides only 16–19% of the daily energy intake. Among the specific foods, the most commonly consumed breakfast foods among children and teenagers were chocolate, pastries and milk [62]. Additionally, a review studied the benefits of breakfast by involving national dietary survey data from various countries including Spain, the UK, Canada, the USA, Denmark and France. Its results found that a healthy regular breakfast has been associated with improved cognitive health, nutritional status and lower plasma cholesterol levels among children and adolescents [63]. These results were supported by a cross-sectional study conducted among 126 children in four elementary schools in Indonesia. Results from that study found that breakfast habits of children were significantly associated with the parent’s breakfast habits [64]. Moreover, in the same study, 23% of fathers and 15.9% of mothers were not having breakfast daily, whereas, 17% of children reported that they are not taking their breakfast because no food was available at home in the morning [64].

One of the most wide-reaching reports is that of the European branch of the World Health Organization, who conducted a health behavior survey of over 200,000 male and female schoolchildren, 11–13 and 15 years of age in 39 European states in 2009/2010 [63]. Overall, 61% of 13-year-olds consumed a breakfast on each school day, while the figure fell to 55% among 15-year-olds. In general, breakfast consumption was most common among boys and declined with lower socio-economic status [63]. These data showed that about half to one third of children do not have breakfast every day, although the data does not reveal the actual frequency of breakfast intake [63]. The report also indicates that regular breakfast consumption is associated with higher intakes of micronutrients, a better diet that includes FV and less frequency of consumption of soft drinks [63]. According to the Health Sponsorship Council (HSC), there are more than 100,000 children worldwide aged 1–5 years missing breakfast at least once per week, while their parents are also skipping this meal. Besides, over 36,000 children worldwide never consume breakfast at home. It has been revealed that children of parents who skip breakfast are more likely to skip their breakfast, consume more energy-dense nutrient-poor food and are more likely to be overweight [65]. A cross-sectional study including 426 children aged 10–14 years from 4 local schools in Queensland found that skipping breakfast among children was associated with the lack of perceived parental emphasis on consuming breakfast (OR = 3.67, 95% CI: 1.75–7.68) [66].

Another cross-sectional survey conducted among preschoolers aged 2–5 years in Hong Kong showed that most children were having their breakfast daily but less than half of them consumed the recommended number of dairy products and FV [31]. Consequently, these studies suggested that parental breakfast-skipping habits are strongly associated with breakfast skipping among their children. Thus, findings underline the importance of addressing parental habits and their children’s in the intervention plan.

### 7.3. Snacking Habits

“Snack” has been defined as a small portion of foods or drinks that is taken between regular meals [67]. Another study considered snacks as food items consumed at different times of the day [68]. A study conducted in Spain defined snacking as the process of consuming any food intake outside the three main meals, including mid-morning snack “between breakfast and lunch” and mid-afternoon snack “between lunch and dinner”, and nibbling, “disorganized and without defined timing” [69]. The term “snack” seems not to have a static definition [67]. Thus, the impact of snacking is difficult to be assessed due to the variety of its definitions in the literature [67].

In Spain, it has been found that 84.4% of younger and 78.3% of older children were mid-afternoon snack consumers. Specifically, sandwich was the most common food item consumed [69]. Excessive consumption of soft drinks and high-fat-containing snacks and low intake of fruits and vegetables was reported among Mexican children in five Baja California counties [70]. Similar findings were found in a cross-sectional study which involved 109 students and their parents in Milan. Results showed that more than 35% of snacks consumed by school-age children were sweets, 23.8% sugary drinks, 9.4% savory snacks, whereas consumption of nuts, yogurt and fresh fruits was very low [71].

Despite limited data, a systematic review concluded that parents’ eating behaviors, whether positive or negative, have an impact on the quality of snacks consumed by their children [72]. Whereas consumptions of lower-quality snacks were associated with increased prevalence of overweight among children [73,74,75]. Some research studies found that the influence of parents on children’s snacking habits depends on the children’s life stage and age. For instance, parental influence decreases in the transition from childhood to adolescence [76,77]. The nationally representative surveys of food intake in US children demonstrated a positive association between parents’ and children’s snack consumption, where children tend to consume more snacks if their parents prefer to have more snacks throughout the day [78]. A cross-sectional study which included 1632 elementary school children in Japan showed that their snacking habits were affected by paternal eating habits, for example, children did not consume vegetables as snacks as it was not offered by their parents. Nonetheless, since data were collected only from children in Takaoka city in Japan, the results may not be generalizable to a global population [79], whereas children’s consumptions of FV as snacks were high in homes with greater FV intake among parents as well as FV availability [79]. These results were confirmed by another study which used a Web-based survey among 9842 students in Australia and found that when parents offered more snacks, children consumed more snacks [80]. Another cross-sectional study conducted among 667 students selected from schools in West-Flanders (Belgium) confirmed that parental monitoring and child’s eating schedule or routine set by parents were associated with more FV intake among girls (*p* ≤ 0.001, 95% CI: −1.8 to −0.5) and boys (*p* ≤ 0.001, 95% CI: −1.7 to −0.5), and reduced negative eating behaviors such as less unhealthy snacking [81]. Results of comprehensive questionnaires, completed by parents of children aged 4–8 years (*n* = 203) in New Zealand, found that the lack of rules regarding the offering of foods to children was associated with a higher intake of fatty snacks [82].

Based on previous studies, it is suggested that during school age, parents play an important role in the control of children’s food intake and food choices. Thus, the whole family is encouraged to be involved in the educational interventions to prevent imbalanced snacking behaviors in children.

## 8. Conclusions

Multiple parental factors influence a child’s dietary habits and are reciprocally interacting, so they cannot be considered separately. The family environment that surrounds a child’s domestic life has an active role in establishing and promoting behaviors that will persist throughout their life. Family meals seem to represent an important moment of both control and interaction, which contributes the most in modeling children’s dietary habits. Parents should avoid excessive pressure or restriction as it can create a negative social and emotional experience that could affect children’s acceptance of the food. Instead, parents should encourage their children on healthy snacking as well as not to skip their breakfast. This can be achieved through positive and active social modeling as well as moderate restriction. Given the considerable evidence for the strong effect of parents on their children’s dietary habits, we believe that parents’ child-feeding behaviors should receive more attention in childhood obesity prevention policies. We recommend that parents should be provided with information and guidance on how, as well as what, to feed their children, and these promotion strategies should be particularly aimed at parents’ unhealthy eating too so they can improve their diet and so their children will imitate them.

## Data Availability

No new data were created or analyzed in this study. Data sharing is not applicable to this article.

## Supplementary Materials

The following are available online at https://www.mdpi.com/article/10.3390/nu13041138/s1, Table S1: Studies assessing the influence of parental dietary behaviors on children’s eating habits.
