# Supplementary material for: The Influence of Parental Dietary Behaviors and Practices on Children’s Eating Habits
Source: Nutrients. 2021 Mar 30;13(4):1138. doi: 10.3390/nu13041138 (PMC8067332; doi:10.3390/nu13041138)
Supplement: Supplementary file 1 [file nutrients-13-01138-s001.pdf]

Table S1. Illustrates some studies assessing the influence of parental dietary behaviors on children's eating habits

Author/year	Country	Participant characteristics	Study design	Main outcome
		**(Longitudinal studies)**		
**Lipsky, L., et al. (2015).** **Ref. [56]**	USA	2785 ethnically diverse American Students	Longitudinal study	Fruit/vegetable intake frequency was associated positively with family meals (β ± SE = 0.33 ± 0.05) and breakfast (0.18 ± 0.03), and inversely with fast food (−0.31 ± 0.04)
**Harbec, M., et al. (2018).** **Ref. [16]**	Australia	1492 children Age 6 and 10 years	Longitudinal study	Children who had a better family meal environment at age of 6 years old, showed lower levels of soft-drinks consumption and higher levels of fitness when children reached 10 years of age
**Galloway, A., et al. (2005).** **Ref. [36]**	USA	173 (9-year-old) non-Hispanic white girls and their mothers	Longitudinal study	Mothers who were less likely to pressure their children , were consuming more fruits and vegetables had daughters who were less picky and consumed more fruits and vegetables
**Sen, B., (2006).** **Ref. [60]**	USA	5014 ethnically diverse school students (boys and girls)	Longitudinal survey	Positive association between high frequency of family meals and reduced incidence of obesity in the future
**Birch, L., et al. (2003).** **Ref. [35]**	USA	197 girls (ages 5, 7, 9 y) and their parents	Longitudinal data	Restricted parental feeding practice seemed to be related to overeating especially among preschool-age children (P<0.001)

**(Cross-sectional studies)**				
**Utter, J., et al. (2007).** **Ref. [5]**	New Zealand	3245 ethnically diverse school students (boys and girls)	Cross-sectional study	Having meals at home with parents was associated to decreased skipped breakfast along with increased consumption of dairy products, fruits and vegetables (P<0.001)
**Fisher, L., et al. (2000).** **Ref. [38]**	USA	197 girls aged 4.6 to 6.4 years and their parents	Cross-sectional study	When parents highly restrict energy-dense snacks from their children's diet hoping children choose healthful alternatives, children usually increase their desire for it and start to eat when they are not hungry.
**Hennessy, E., et al. (2012).** **Ref. [37]**	USA	Children partner schools (grades kindergarten through five, n83)	Cross-sectional study	Parental eating habits and feeding styles have been associated with children's snacking habits, the intake of dairy, high fat and sugar, fruits and vegetables
**Cheng, T., et al. (2008).** **Ref. [66]**	Australia	426 students aged 10-14 years (Boys & girls)	Cross-sectional study	Skipping breakfast among children were associated with the lack of perceived parental emphasis on breakfast
